# Diagnosis and Treatment of Biliary Fistulas in the Laparoscopic Era

**DOI:** 10.1155/2016/6293538

**Published:** 2015-12-24

**Authors:** M. Crespi, G. Montecamozzo, D. Foschi

**Affiliations:** II Unit of General Surgery, Luigi Sacco Hospital, Department of Biochemical Sciences L. Sacco, University of Milan, Via G.B. Grassi 74, 20157 Milan, Italy

## Abstract

Biliary fistulas are rare complications of gallstone. They can affect either the biliary or the gastrointestinal tract and are usually classified as primary or secondary. The primary fistulas are related to the biliary lithiasis, while the secondary ones are related to surgical complications. Laparoscopic surgery is a therapeutic option for the treatment of primary biliary fistulas. However, it could be the first responsible for the development of secondary biliary fistulas. An accurate preoperative diagnosis together with an experienced surgeon on the hepatobiliary surgery is necessary to deal with biliary fistulas. Cholecystectomy with a choledocoplasty is the most frequent treatment of primary fistulas, whereas the bile duct drainage or the endoscopic stenting is the best choice in case of minor iatrogenic bile duct injuries. Roux-en-Y hepaticojejunostomy is the extreme therapeutic option for both conditions. The sepsis, the level of the bile duct damage, and the involvement of the gastrointestinal tract increase the complexity of the operation and affect early and late results.

## 1. Introduction

Biliary fistulas are defined as chronic pipe-like ulcers. They can connect the gallbladder with the biliary tree and rarely involve the gastrointestinal tract (internal fistulas) and the abdominal wall (external fistulas) [1]. Biliary fistulas are rare complications of lithiasis or neoplasia and are classified as primary and secondary [2].

Internal fistulas are always caused by inflammation and occur mainly as late complications of gallstone or hydatid diseases, like biliobronchial fistulas [3].

External fistulas are related to the iatrogenic injury of the biliary tract and are infrequent compared to primary fistulas.

The incidence of the primary biliary fistulas is ranged from 1 to 2%, in symptomatic patients; in Latin America it is more common (4.7–5.7%) [4]. The widespread use of ultrasonography and the early treatment for patients with gallstone disease with laparoscopic surgery reduce the incidence of biliary fistulas. However, the laparoscopic cholecystectomy has slightly increased the secondary fistulas in comparison with open surgery (0.3–0.4% to 0.6%) [5]. The overall incidence of laparoscopic complications is related to surgical experience: 90% of the injuries occur in the first 30 cases, with a reduction from 1.7% to 0.17% after the 50th case [6]. The use of new laparoscopic techniques (i.e., single port surgery) seems to be associated with a higher rate of injuries, probably by the necessity of a new learning curve [7].

The complication rate of open cholecystectomy has increased for two reasons: the overall declining experience in the open approach and its use only in challenging cases [8].

Since only 20–30% of the patients affected by gallstone are symptomatic [9], the diagnosis in the early stage is not easily recognizable. In the late stage, the clinical presentation is tricky, considering that the main symptoms and signs are various:

- jaundice exists when stricture of the bile duct is associated with the fistula (Mirizzi's Syndrome);
- cholangitis and sepsis exist when bacterial overgrowth is associated with the inflammation of the gallbladder and the biliary tract;
- bowel occlusion occurs when the passage of large stones in the alimentary tract causes obstruction of the small bowel, usually in the terminal ileum (Bouveret's syndrome);
- derangement of hepatic function tests is variably present;
- aerobilia, at the Rx abdominal plain or CT, is a pathognomonic sign of biliary fistulas.

## 2. Physiopathology and Classification

### 2.1. The Primary Biliary Fistulas

Kehr was the first who described gallstone obstruction of the hepatic duct in 1905 [10]. In 1948, Mirizzi described a case of hepatic duct compression secondary to an impacted gallstone in the infundibulum of the gallbladder; this clinical condition was named “Mirizzi's Syndrome” [11]. This is the first stage of the pathways leading to biliary fistulas.

**Figure 1:**
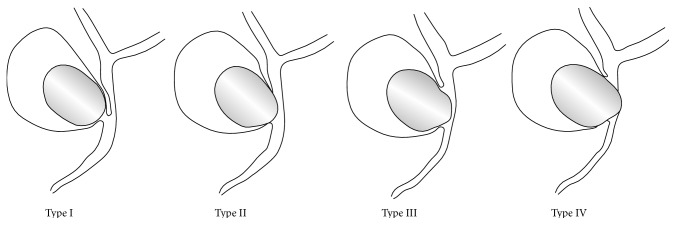
Classification of the Mirizzi's Syndrome by Csendes.

In 1942, Puestow [12] reported a series of 16 patients, with a spontaneous internal biliary fistula between the gallbladder, the choledochus, and other abdominal and thoracic organs. In 1989, Csendes et al. proposed a new classification of patients with Mirizzi's Syndrome. Fistulas from the gallbladder to the common bile duct or the hepatic duct are defined as evolving stages of the same disease [13] (Figure 1).

- Type 1 lesion is the external compression of the common bile duct due to a gallstone impacted at the neck of the gallbladder or at the cystic duct (the original Mirizzi's syndrome).
- Type 2 lesion is the presence of a cholecystobiliary fistula (cholecystohepatic or cholecystocholedochal) due to the erosion of the anterior or lateral wall of the common bile duct by impacted stones; the fistula involves less than one-third of the circumference of the common bile duct.
- Type 3 lesion is the presence of a cholecystobiliary fistula with erosion of the wall of the common bile duct that involves up to two-thirds of its circumference.
- Type 4 lesion is the presence of a cholecystobiliary fistula with complete destruction of the entire wall of the common bile duct.

This physiopathological process begins with the impact of the stones and continues with the erosion of the gallbladder and the common bile duct wall. The fistula can involve the biliary tract and nearby gastrointestinal structures. Based on this physiopathological process, cholecystoenteric fistulas must be considered the late evolving stages of the Mirizzi's Syndrome.

In 2008, Beltran et al. [14] proposed the inclusion of the cholecystoenteric fistulas in the Mirizzi's Syndrome's classification as type 5: every type of lesion, plus cholecystoenteric fistula, without gallstone ileus (5a), and with gallstone ileus (5b). Bilioenteric fistulas are classified as [14]

- cholecystoduodenal fistulas: 40%;
- cholecystocolic fistulas: 28%;
- cholecystogastric: 32%.

Large stones, recurrent cholangitis, female sex, and old age are risk factors for bilioenteric fistulas [15]. In the absence of stones, a bilioduodenal or more complex fistula can be caused by peptic ulcer or hydatid disease.

### 2.2. The Secondary Biliary Fistulas

Secondary biliary fistulas are caused by iatrogenic injury during cholecystectomy, either performed by open or laparoscopic surgery. The main condition favoring injury is an unclear anatomy of the biliary tract due to local peritonitis, inflammation, or bleeding during the operation. The failure to identify the anatomical landmarks within the Calot's triangle is the most frequent reason of the bile duct injury [6]. Although the study of the biliary tract with intraoperative cholangiography has been considered advantageous to avoid injury, its frequent use is not recommended [16, 17]. Intraoperative laparoscopic ultrasound has been proposed as an alternative way to study the biliary tract, with an accuracy of 94–96% [18]. As a whole, the incidence of secondary biliary fistulas is low (0.3–0.6% of all cholecystectomies); the clinical presentation is characterized by bile leakage in the abdominal cavity. If a drain is in the subhepatic space, an external fistula develops. Without drainage biliary peritonitis is found. In the 80s, Bismuth proposed a classification of the iatrogenic injuries of the biliary tract based on the level of transection from the confluence of the hepatic ducts [19]:

 type I: transection > 2 cm from the confluence;
 type II: transection < 2 cm from the confluence;
 type III: transection in the hilum;
 type IV: separation of the major ducts in the hilum;
 type V: transection injury of aberrant right hepatic duct plus injury in the hilum.

This classification refers to open surgery and it is very useful to plane the surgical operation, but it does not consider the mechanisms leading to biliary duct damage during laparoscopic cholecystectomy. Wrong clipping of the cystic duct or thermal injury by cautery in dissecting Calot's structures may cause lateral damage of the bile ducts. The classification of the injuries into 5 types proposed by Schmidt et al. allows us to distinguish between lateral damage and complete section or closure of the bile duct [20]. Patency of the cystic duct or leakage from the liver bed and lateral incomplete section, or clipping, can be treated by endoscopic measures. The decision depends also on the output of the leakage: low output leakage (<100 mL/day) from cystic duct or Luschka's in the bed liver usually goes to resolution spontaneously in less than 30 days [21, 22].

When the output of the fistula is high (usually >100 mL/day for few days), endoscopic treatment is indicated to avoid a future stenosis. Major damage on the bile ducts (i.e., complete transection) should be treated by a surgeon with a sufficient experience in the advanced biliary surgery.

## 3. Diagnosis

The preoperative diagnosis of the biliary fistulas is challenging and it is achieved only in 8–17% of the cases [4].

To plan the best operation, we need to know

- the cause of the fistula: the presence of gallstones is the most frequent pathological condition; in the absence of stones, other inflammatory conditions or neoplasia should be searched for;
- the level of obstruction of the biliary tract and the presence of erosion/destruction of the wall;
- the presence of bilioenteric fistula, easily recognizable by aerobilia and/or biliary ileus.

The presence of gallstones is easily demonstrated by noninvasive diagnostics.

Transabdominal US has a sensitivity of 96% regarding gallstones detection [23]. A partial obstruction by external compression of the common bile duct and a normal distal common bile duct are anatomic features frequently associated with Mirizzi's Syndrome (MS). The diagnostic accuracy for transabdominal US in (MS) is 29% [4].

**Figure 2:**
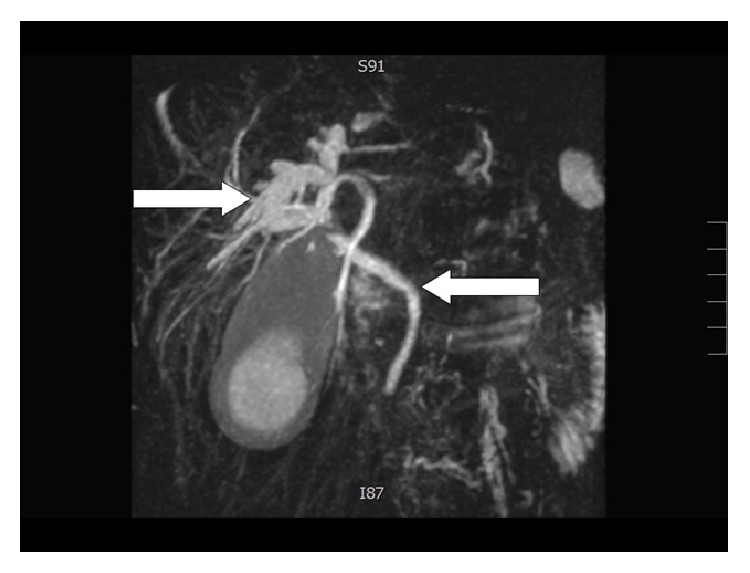
Dilatation of the intrahepatic bile ducts with a normal choledochus (personal observation).

Magnetic resonance cholangiopancreatography (MRCP) has a better diagnostic accuracy (about 50% of the cases) and provides better information of the anatomy of the biliary tree and the gallbladder [24] (Figure 2).

**Figure 3:**
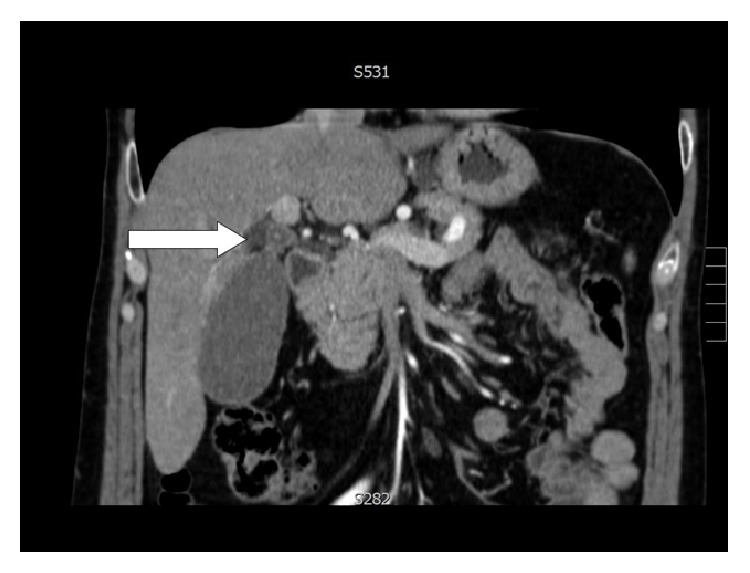
Level of obstruction (personal observation).

The level of obstruction is easily demonstrated by CT, which is also useful to exclude neoplastic lesions located at the hepatic hilum or into the liver [4, 9] (Figure 3).

**Figure 4:**
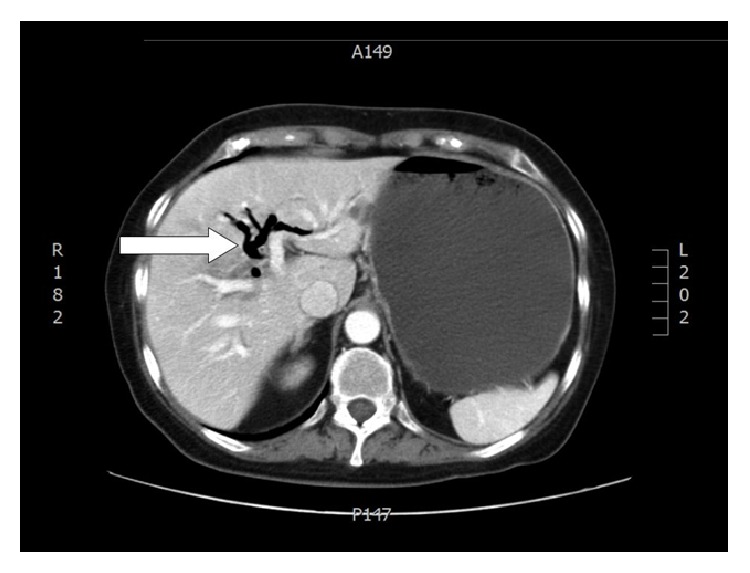
Aerobilia (personal observation).

**Figure 5:**
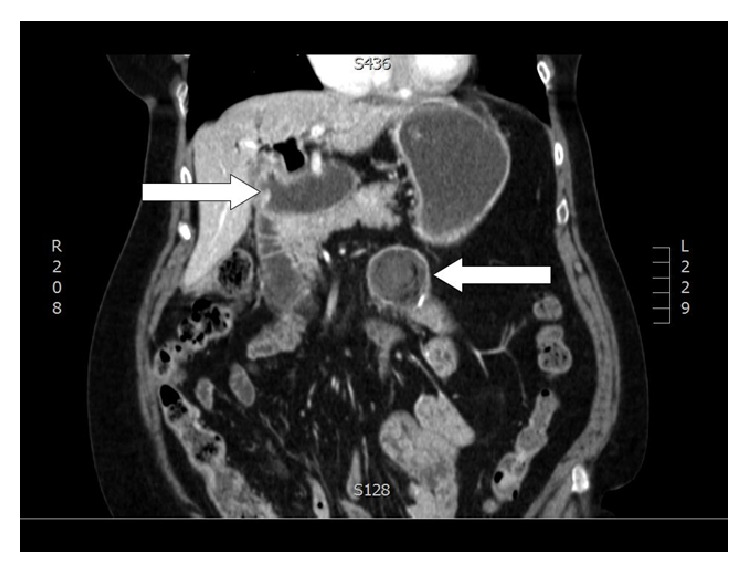
Duodenal fistula and bowel obstruction (personal observation).

Aerobilia is evident using either US or CT. Bowel obstruction by a biliary stone is typically evident in the CT scans (Figures 4 and 5).

Invasive procedures, like endoscopic ultrasound (EUS) and endoscopic retrograde cholangiopancreatography (ERCP), have a higher diagnostic accuracy [25]. There are only few reports on the diagnosis of biliary fistulas by EUS, but it has been suggested that intraductal ultrasonography (IDUS) has a high sensitivity (97%) and specificity (100%) in all types of Mirizzi's Syndrome [26–28]. ERCP has a diagnostic accuracy of the primary biliary fistulas ranging from 55% to 90% and it has also a therapeutic-operative role: retrieval of the stones after sphincterotomy and placement of stents and a nasobiliary drainage of the biliary tree are useful for the best treatment of the patients [29, 30].

### 3.1. Secondary Biliary Fistulas

The diagnosis of biliary injury can be reached during cholecystectomy, performed with an opening of laparoscopic surgery. The use of intraoperative cholangiography is useful to identify the site and size of the damage, the presence of common bile duct stones, the presence of stenosis, and other pathological conditions. The correct intraoperative diagnosis allows us to correct the damage immediately, without inflammation and peritonitis [31, 32]. When a difficult cholecystectomy is performed, the use of drain in the subhepatic space is useful to identify a minor bile leak promptly. Without a drain, a complicated postoperative course (nausea, vomiting, fever, abdominal pain, and jaundice) requires immediate investigation. US, CT, and MRCP are used to identify the site and size of the biliary lesion and the presence of stones in the common bile duct.

ERCP is useful both for diagnosis (accuracy 95%) [33] and for therapy. Finally, percutaneous transhepatic cholangiography should be applied for patients with complete closure of the distal biliary duct to define the level of the lesion. In selected cases, percutaneous transhepatic drainage can be performed to drain the biliary tract.

## 4. Treatment

### 4.1. Primary Biliary Fistulas

The surgical treatment of primary biliary fistulas is a challenge for surgeons.

A good knowledge of the pathology, the damage on the biliary tree, and the involvement of the alimentary tract is necessary. The inflammation in Calot's triangle causes a significant derangement of the anatomy of the hilum region and may expose the surgeon to intraoperative injury of the bile duct.

Laparoscopic surgery can be applied to type I Mirizzi's Syndrome and treatment of biliary ileus [34]. The retrograde cholecystectomy is the classic approach, but an anterograde surgery can be used for difficult cases. Some authors proposed a subtotal cholecystectomy as a further option for these patients [35, 36]. If you need to explore the common bile duct, it is better to make a separate incision that can be also used to drain the biliary tract with a T tube.

In type 2 Mirizzi's Syndrome, where a limited involvement of the biliary tract is present, the operation should include a subtotal cholecystectomy, leaving a remnant gallbladder wall (5 mm in size) to perform the reconstruction of the bile duct. The drainage of the bile duct by T tube is performed to protect the choledocoplasty. Laparoscopic surgery is difficult, and it is made only in favourable conditions. Robotic surgery, consisting of subtotal cholecystectomy, associated with plastic stent insertion at ERCP, has been successful in a personal limited series [37].

In type 3 fistulas, the best treatment is the subtotal cholecystectomy with choledocoplasty, but an hepaticojejunostomy should be also considered when the damage is large, as in type 4 fistulas [4, 38]. The operation needs an open surgery.

In type 5 fistulas, the presence of biliary ileus allows for an emergency treatment; in the absence of septic complications, the operation consists of enterotomy and stone extraction (it can be performed laparoscopically) with delayed treatment of the cholecystoenteric fistula. When septic complications occur, the operation needs also the treatment of the fistula [39].

Since no large series have been described, the surgical treatment should be proposed based on personal experience; there is no scientific evidence for the best surgical treatment.

From the systematic review of Antoniou et al. [40], laparoscopic cholecystectomy is performed mainly in type 1 Mirizzi's Syndrome; the presence of a fistula is considered by many surgeons a contraindication to laparoscopy. However, the conversion rate to open surgery is the same whether compression (47%) or fistulas (43%) are present [33]. The complication rate is slightly higher after treatment of type 2 (19.3%) rather than type 1 (16.2%) Mirizzi's Syndrome. Bile duct injury and residual stones are the most frequent complications. There is a significant correlation between the accuracy of the preoperative diagnosis and the rate of conversion, complications, and reoperations. Laparoscopic treatment of Mirizzi's Syndrome is possible and safe only if the operation is planned on the basis of the knowledge of the anatomical and pathological conditions.

### 4.2. The Secondary Biliary Fistulas

Most of the low grade leaks occur from a cystic duct or Luska's and can be treated definitively by an endoscopic approach. The aim is to decrease the transpapillary pressure gradient; a good transpapillary bile flow allows for a reduction of the biliary loss from the leakage [41, 42]. The insertion of a biliary stent across the papilla without sphincterotomy is generally desirable to preserve the biliary sphincter, particularly in younger patients. Sphincterotomy must be done only in case of common bile duct obstruction secondary to choledocolithiasis in order to remove the retained stones or in case of a high output leak. The patient requires a biliary stent since sphincterotomy does not always completely eliminate the transpapillary pressure gradient.

The stent is left in place for approximately four to six weeks and removed if ERCP shows the resolution of the leakage. The same approach can be used for minor lateral injuries of the right or common bile duct.

When surgery is necessary, it is usually undertaken to drain loculated collections rather than repair defects in the continuity of the biliary tree. In 10% of patients, bile leaks do not respond to sphincterotomy and/or plastic stent placement [43]: such cases can be managed by temporary placement of a covered, self-expanding metal stent [44].

In the case of refractory bile leaks, we must keep in mind the possibility that the lesion is coming from transection of an anomalous aberrant right hepatic duct from which the cystic duct arose. Diagnosis may require MRCP; this lesion often required a surgical operation involving preferably a hepaticojejunostomy. Injuries to main common bile or common hepatic ducts are the most serious and are similar to the injuries most commonly seen in open cholecystectomy [5]. Clinical conditions are highly variables and can deteriorate rapidly, depending on the type of injury: the main duct may be completely transected or clipped with or without bile leak. The patients with bile leak have early symptoms (sepsis and peritonitis) with a median of three days, while patients developing stricture without bile leak have a significant longer symptom-free interval. Early diagnosis can be obtained by US and CT scan; MRCP is useful to define biliary anatomy particularly in patients who preclude ECRP by complete biliary transection. The presence of concomitant right hepatic artery injury should be assessed, since it is a prognostic factor of late complications. Primary surgical repair of the bile ducts, in the presence of an acute local inflammatory response, should be avoided because of the high rate of breakdown or stricture formation. Injuries over the biliary bifurcation cause high risk of early and late complications; the surgery involves a bilioenteric anastomosis in all cases, usually a proximal hepaticojejunostomy with a Roux-en-Y jejunal for the prevention of ascending cholangitis. These operations can be difficult and time-consuming. Consequently, any complex biliary injury recognized at the time of operation by a surgeon with minimal experience in complex biliary reconstruction should not be repaired at that time. Instead, the patient should be stabilized and transferred as soon as possible (better within 24 hours) to an institution with hepatobiliary expertise.
